# Cardiac Development and Factors Influencing the Development of Congenital Heart Defects (CHDs): Part I

**DOI:** 10.3390/ijms25137117

**Published:** 2024-06-28

**Authors:** Marek Zubrzycki, Rene Schramm, Angelika Costard-Jäckle, Jochen Grohmann, Jan F. Gummert, Maria Zubrzycka

**Affiliations:** 1 Department of Surgery for Congenital Heart Defects, Heart and Diabetes Center NRW, University Hospital, Ruhr-University Bochum, Georgstr. 11, 32545 Bad Oeynhausen, Germany; mzubrzycki@hdz-nrw.de; 2 Clinic for Thoracic and Cardiovascular Surgery, Heart and Diabetes Center NRW, University Hospital, Ruhr-University Bochum, Georgstr. 11, 32545 Bad Oeynhausen, Germany; rschramm@hdz-nrw.de (R.S.); ajaeckle@hdz-nrw.de (A.C.-J.); jgummert@hdz-nrw.de (J.F.G.); 3 Department of Congenital Heart Disease/Pediatric Cardiology, Heart and Diabetes Center NRW, University Hospital, Ruhr-University Bochum, Georgstr. 11, 32545 Bad Oeynhausen, Germany; jgrohmann@hdz-nrw.de; 4 Department of Clinical Physiology, Faculty of Medicine, Medical University of Lodz, Mazowiecka 6/8, 92-215 Lodz, Poland

**Keywords:** cardiac development, cardiogenesis, heart morphogenesis, sequential segmental analysis, etiology, genetic factors, congenital heart defects

## Abstract

The traditional description of cardiac development involves progression from a cardiac crescent to a linear heart tube, which in the phase of transformation into a mature heart forms a cardiac loop and is divided with the septa into individual cavities. Cardiac morphogenesis involves numerous types of cells originating outside the initial cardiac crescent, including neural crest cells, cells of the second heart field origin, and epicardial progenitor cells. The development of the fetal heart and circulatory system is subject to regulatation by both genetic and environmental processes. The etiology for cases with congenital heart defects (CHDs) is largely unknown, but several genetic anomalies, some maternal illnesses, and prenatal exposures to specific therapeutic and non-therapeutic drugs are generally accepted as risk factors. New techniques for studying heart development have revealed many aspects of cardiac morphogenesis that are important in the development of CHDs, in particular transposition of the great arteries.

## 1. Introduction

The development of most heart defects takes place not in neonates but in the early prenatal period. Therefore, it is important to have reliable knowledge of cardiac embryology and fetal circulatory system physiology, which allows to understand the changes occurring within the heart in newborns. Over the past decade, thanks to various discoveries, we have become better acquainted with the development of the primordium of the heart. The development of the circulatory system begins with the early separation of bilateral clusters of progenitor cells, which coalesce to form a cardiac crescent, followed by a linear heart tube in the midline of the body. That tube, consisting of an inner layer of endothelial cells surrounded by myocardial precursor cells, forms subsequently a series of loops and bends, followed by a balloon-like enlargement of the areas from which the heart cavities are to be formed [1,2,3]. Later, the cardiac septa develop, which results in the formation of a four-cavity organ with a separate, parallel systemic and pulmonary circulation [4,5,6]. Additional types of cells found outside the heart tube play an important role in the development of the heart and affect its morphogenesis. For example, neural crest cells, which form the building blocks of the peripheral nervous system and craniofacial areas, migrate to the heart, where they are indispensable for the formation of a septum within the outflow tract [6,7,8]. The relationship between neural crest cells and the formation of septa in the heart explains the correlation between craniofacial defects and some congenital heart defects [9]. In heart development, the critical contribution of precursors originating outside the primitive heart tube may explain the co-occurrence of congenital heart defects (CHDs) and extracardiac anomalies in patients with genetic syndromes. Until recently, the origin and development of these different cell types were unclear. Recently, however, new techniques for studying the development of the heart and gene targeting have made it possible to “track the fate of the cells” as well as to analyze cell lines in embryos and adult mammals [3]. Studies have shown that heart development is controlled by several overlapping morphogentic systems that regulate intricate cardiac transcriptional networks. Nodal, bone morphogentic proteins (BMPs), WNTs, Sonic hedgehog (SHH), NOTCH, neuregulin, retinoic acid, fibroblast growth factors (FGFs), and other signaling molecules are known to play essential roles in regulating cardiogenesis, primarily by controlling cardiac transcription factors MESP1, GATA4, NKX2-5, HAND1, HAND2, ISL1, and several TBX transcription factors [10,11,12]. 

During the past decades, a consensus has emerged that both genetic factors (e.g., chromosomal abnormalities, smaller copy number variants, and point mutations) and environmental ones (extrinsic factors, such as maternal smoking during pregnancy, teratogen exposure, and nutrient deficiencies; intrinsic factors, including maternal disease, gestational diabetes, and viral infections) are related to the occurrence of CHDs [13,14,15,16]. 

A promising approach for the identification of essential cardiac regulators whose mutations may be linked to human transposition of the great arteries is the molecular and genetic analysis of heart development. Epigenetic factors have been underestimated in their influence on cardiac development [11,17,18,19]. Advances in the understanding of cardiac development are likely to have an even greater impact on the classification and treatment of congenital heart defects. Thanks to this, it will also be possible to diagnose defects at a very early stage, which in turn will make it possible to counteract their natural course, or to implement treatment at the earliest possible stage. 

## 2. Stages of the Human Heart Development

Knowledge of the normal formation of the heart is crucial for understanding cardiac pathologies and congenital malformations. 

**Figure 1:**
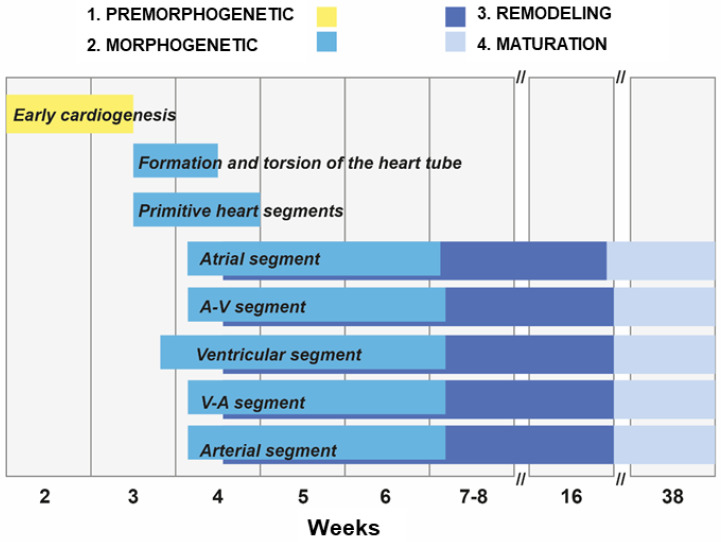
Stages of the human heart development. A-V: atrioventricular; V-A: ventriculoatrial. Numerical data for the figure was taken and adapted from Villavicencio-Guzman et al. [3], under the terms of the Creative Commons Attribution License (CC BY).

Heart development is a multistage, dynamic, sequential, morphologically ordered, and genetically and epigenetically regulated process [4,5,6,20,21,22]. It is usually divided into four stages that overlap in time (Figure 1) [2,3]. 

These are:

1. Early cardiogenesis: It occurs during the premorphogenetic or presomitic stage of the embryo (days 8–18 of development). Early cardiogenesis begins with the organization of the cardiac areas and crescent through gastrulation and ends with the formation of two endocardial tubes that are externally covered by myocardial lineage cells.
2. Morphogenetic stage: This stage occurs during weeks 4–8 of embryonic development. It begins with the formation of the straight heart tube, derived from the first heart field (FHF), and ends after the integration of the primordia of all the structures that comprise the four-chambered heart, derived from the second heart field (SHF).
3. Septation and remodeling of the heart chambers: This stage begins during midembryonic development (day 30). At this stage, the primordia undergo differential growth and remodeling processes. The valves and septum are formed, and concurrently, the atrial and ventricular cavities acquire their morphological identities.
4. Maturation and histodifferentiation: It occurs during the fetal period (weeks 16–38) and involves histological maturation of the ventricular and atrial myocardium and histological differentiation of the ventriculoarterial and atrioventricular valve systems, including the tendinous cords and papillary muscles. Concurrently, the conduction system and coronary vessels are developed [3].

The cells involved in heart development come from different cardiac cell populations, called heart fields, and other non-cardiac cell populations, such as neural crest and pre-epicardial organ cells [2,3,22,23,24,25]. Each of these populations plays a crucial role in the development of the cardiovascular system. Any perturbation in the cells that contribute to the building of the heart leads to cardiac malformations, which frequently result in the death of the embryo [26].

## 3. Chronology of the Development of the Heart

**Figure 2:**
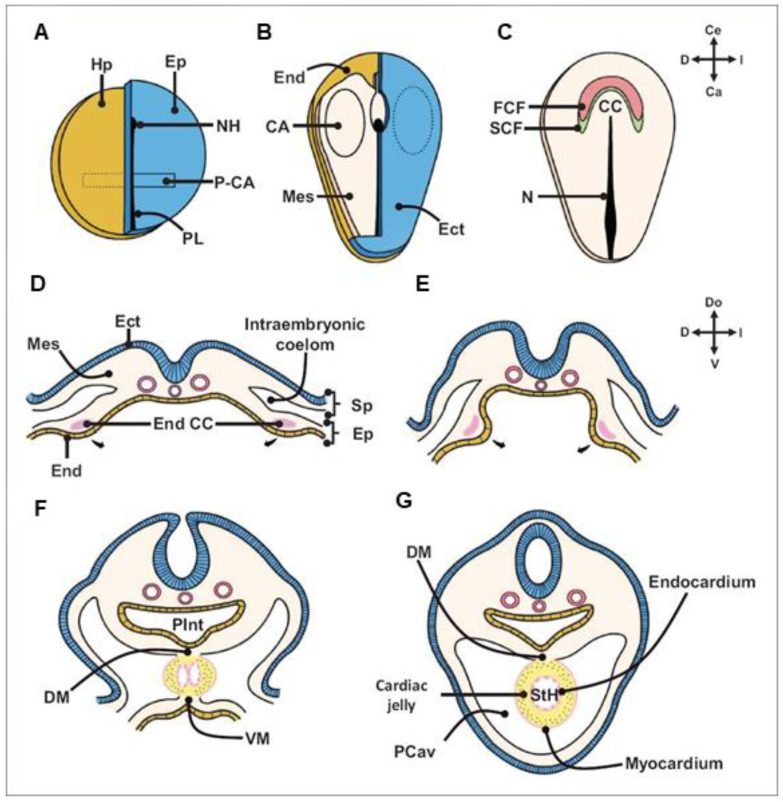
Early cardiogenesis. (**A**) blastula. Two pre-cardiogenic areas are present in the epiblast. (**B**) early gastrula. The pre-cardiogenic areas migrate through the primitive streak to incorporate into the splanchnic mesoderm, forming the cardiogenic areas. (**C**) late gastrula. The cardiogenic areas migrate in a cephalomedial direction and fuse to form the cardiogenic crescent. (**D**) the cardiogenic crescent is mobilized in the splanchnopleura, showing its ends as two endocardial tubes. (**E**–**G**) the ends of the cardiogenic crescent move in a ventro-medial direction until they fuse and form a single myo-endocardial tube, called a straight-tube heart. Note that the dorsal wall of the heart is attached to the ventral wall of the primitive gut tube. CA: cardiogenic areas; DM: dorsal mesocardium; Ect: ectoderm; End CC: cardiogenic crescent endings; End: endoderm; Ep: epiblast; FHF: first heart field; Hp: hypoblast; Mes: mesoderm; N: notochord; NH: node of Hensen; P-CA: precardiogenic areas; PCav: pericardial cavity; PGT: primitive gut tube; PS: primitive streak; SHF: second heart field; Sp: somatopleure splanchnopleure; TH: straight-tube heart; VM: ventral mesocardium. This figure is reproduced with permission from Flores et al. [22].

The first signs of cardiogenesis appear at the blastula stage [2,3,22]. Cardiogenesis begins at the end of the gastrula stage, when the embryo separates into the three distinct germ layers: endoderm, mesoderm, and ectoderm. Cardiac precursor cells are the first ones to undergo gastrulation: they migrate laterally and anteriorly from the primitive streak and settle on both sides of the embryo in the lateral plate splanchnic mesoderm, initially forming 2 cardiogenic fields and then a crescent-shaped plate (cardiac crescent) from which the heart tube will be formed (Figure 2) [22,27]. 

The population of cells that make up the cardiac crescent is considered to be the first (primary) heart field (FHF) and contains precursor cells for 2 cell lines: cardiocytes and endocardial endothelial cells, separated by an extracellular matrix called cardiac jelly [3,25,28,29]. FHF cells initiate the formation of the atria of the heart, the atrioventricular canal, the left ventricle, and, to a lesser extent, are involved in the development of the right ventricle and the ventricular outflow tract.

The second component of the nascent heart are the cells of the second (secondary) heart field (SHF), maturing later, initially directly adjacent on medially to the formed primary field, and as the embryo develops, they move towards the dorsal and cephalic parts of the primary heart tube [3,30,31]. These cells have been demonstrated to be involved in the development of the right ventricular wall (the one that does not originate from the first heart field), the conduction system, the outflow tract, the primary venous sinus, the pulmonary veins, as well as the cardiac veins, including the coronary sinus and coronary arteries, and the pre-epicardial organ [23,27,28,30,31,32,33,34,35]. From the very beginning, the cells of the cardiogenic fields are determined to form the right- and left-sided structures of the heart [2].

An additional source of the formation of cardiac structures is the “heart pool” of neural crest cells (CNCCs), which are not of mesodermal origin but are derived from the neuroectoderm. The cardiac neural crest cells, which migrate towards the primary heart tube, are involved in the formation of the arteries of the pharyngeal arches, the outflow tract of the right ventricle, the cardiac ganglia, and together with the pre-epicardial organ, also of the conduction system of the heart [36,37,38]. 

There is still no consensus on the boundaries and fate of the heart fields [3]. Villavicencio et al., have proposed a segmental model of the heart where they suggest that the straight-tube heart is composed of the primordia of the interventricular septum, the left ventricle, and the atrioventricular canal (A-V canal) [3]. Previously, the myocardium was thought to be derived from a single source of cells. However, the recent identification of a second source of myocardial cells that make an important contribution to the structure of cardiac chambers has modified the classical view of heart formation.

## 4. Cardiac Loop Formation

**Figure 3:**
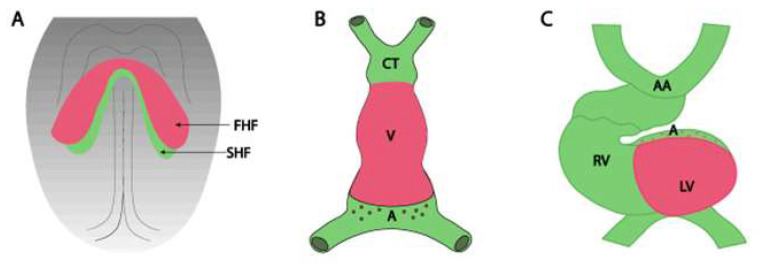
The schematic representation of the cardiac loop formation. (**A**) the configuration of the cardiac crescent, (**B**) the early cardiac straight tube, (**C**) the rightward looping (**C**). FHF: first heart field; SHF: second heart field; CT: conotruncus; V: ventricle; A: atrium; RV: right ventricle; LV: left ventricle; AA: aortic arches. This figure was taken from the article of Lozano-Velasco et al. [39] distributed under the terms of the Creative Commons Attribution License (CC BY).

As a result of bending of the embryo body walls and the fusion of the two initial heart tubes, a single heart tube with bifurcated ends, extending towards the cephalic and caudal sides of the embryo, is formed (Figure 3). A fully formed primary heart tube is shaped like an inverted Y. The heart tube can be divided initially into three parts: the atrium, the ventricle, and the bulbus cordis, and then into 5 individual segments resulting from the heart tube strictures [2,4]. Within the cephalic end, the heart cavity is divided into the primary atrium, the primary ventricle (the inflow part), the primary bulbus cordic (the outflow part), and the venous sinus located in the caudal part. Within the caudal end, the two tubes do not connect completely, and the horns (arms of the letter Y) arising from the venous sinus are reached bilaterally by the outlets of the umbilical veins, basal (common), and yolk veins.

Then, the heart tube grows in length and undergoes a looping process to form the cardiac loop [4]. In the 4th week of embryonic development, the primary heart tube undergoes rotation (clockwise in typical development), in which it is possible to distinguish between protrusions constituting the bulbus cordis and the ventricle. Between them, the tube is slightly narrowed by the ventriculobulbar fold, which then, by bending, forms a ventriculobulbar groove. Subsequently, the ventricular part of the tube loops (cardiac loop) and is divided (septation) [40]. 

Looping of the heart tube is quite complex and involves several stages. In the first phase, as a result of right-sided rotation, the organ takes on the shape of the letter “C”, in which the primary atrium and venous sinus are located dorsally. In the second phase, a left-sided rotation takes place at the height of the so-called ventriculobulbar loop. It is noteworthy, however, that the second rotation is limited only to the primary ventricle, and as a result, the heart takes the shape of the letter “S”. The S-shaped heart loop undergoes a series of differentiation-proliferation cycles to balloon and form the heart chambers [41]. Looping determines the position of the ventricle in relation to the atria. The heart tube may loop to the right (D-loop) or loop to the left (L-loop) (Figure 3). In the case of a D-loop, the morphologic right ventricle lies to the right of the left ventricle, whereas in the case of an L-loop, the morphologic right ventricle is positioned to the left of the left ventricle [42]. Disturbances at this stage of development lead to the formation of many congenital defects, e.g., abnormal rotation of the outflow tract causes transposition of the great blood vessels, i.e., a situation in which the aorta arises from the morphologically right ventricle and the pulmonary trunk from the morphologically left ventricle [43]. 

When the heart bends, protrusions are formed on the ventral surface of both horns of the venous sinus, which grow towards the pericardial cavity called the pre-epicardium [44]. As the pre-epicardium grows, it reaches the surface of the heart, where it forms a layer of epithelial cells, which becomes the epicardium. Cells of the pre-epicardium, and thus of the epicardium, are the precursor material for many structures of the heart: tunica media smooth muscle cells and coronary adventitia fibroblasts, interstitial and valvular fibroblasts of the heart, and a small population of cardiomyocytes. Previously, it was believed that the cells of the pre-epicardium, and thus of the epicardium, are the precursor material for many structures of the heart: tunica media smooth muscle cells and coronary adventitia fibroblasts, interstitial and valvular fibroblasts of the heart, and a small population of cardiomyocytes. According to the current state of knowledge, their contribution to the myocardium has not been demonstrated. Recently, high expectations concerning the epicardium as a candidate source of cells for the repair of the damaged heart have been raised. Because of its developmental importance and therapeutic potential, current research on this topic focuses on the complex signals that control epicardial biology [44]. 

All these cellular components are formed as a result of epithelial-mesenchymal transformation (EMT), which consists in the invagination of epithelial cells into the subepicardium and their transformation into mesenchymal cells, and then into differentiated cells of separate cell lines [22,45]. However, numerous studies propose that this process is regulated by a complex network consisting of TGF-β/Smad, BMP, Wnt/β-catenin, Notch, and Smad-independent TGF-β signaling pathways, which together induce the expression of transcription factors, such as Snail, Slug, and Twist, and promote or inhibit EMT [22,45].

## 5. Differentiation and Remodeling of the Cardiac Chambers

When the looping process is completed, the primary heart tube takes the form of a finally formed heart. In the upper—cephalic—part, the arterial trunk can be distinguished, surrounded by atrial appendages, in the caudal segment—the ventricles of the heart. 

**Figure 4:**
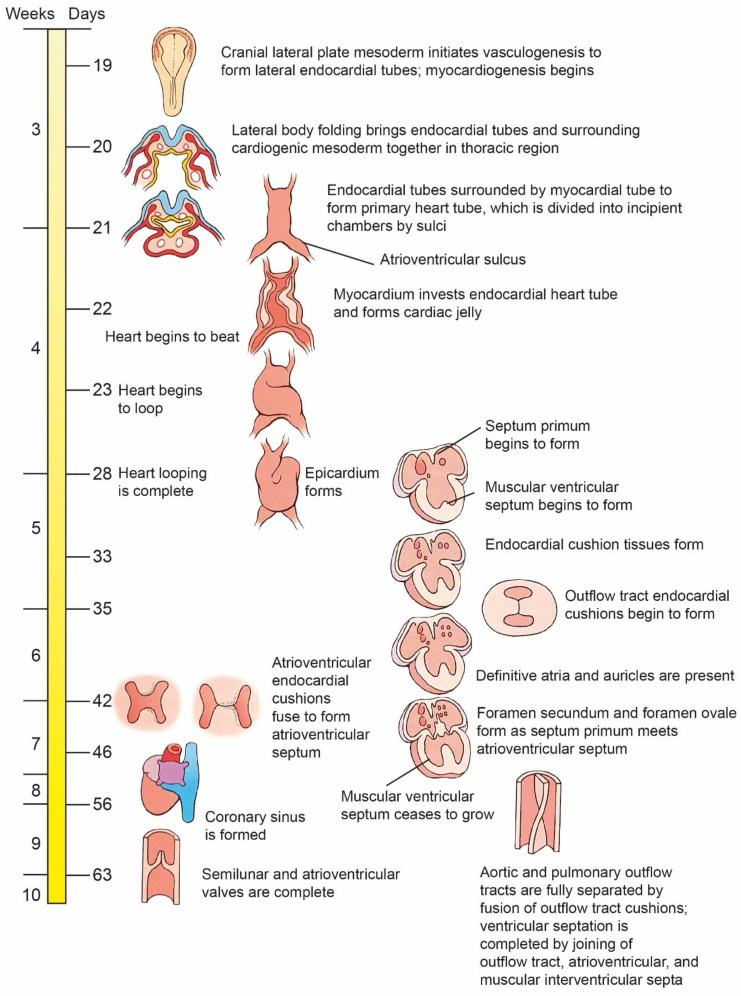
Time line. Formation of the heart. Day 19—formation of the heart tube; day 20—twisting of the tube around its own axis; day 22—the first contractions of the heart tube; days 28–35—internal divisions of the heart tube structures; days 37–44—completion of the development of the heart tube. This figure was modified and adapted from the work of Schoenwolf et al. [46].

As the heart tube increases in length beyond the cardiac loop, internal divisions develop from the endocardial folds at the same time. This process begins between the 4th and 5th week of fetal life, more precisely between the 26th and 37th day of gestation (Figure 4) [2,5,40].

At the level of the atria, the septa are formed: the first one—the primary septum (septum primum)—develops towards the atrioventricular orifice, gradually closing the first opening—ostium primum (a temporary opening in the lower part of the septum) and dividing the atria into the right and left parts. Subsequently, the upper part of the primary septum disappears, and a secondary opening (ostium secundum) is formed to enable the blood flow from the right to the left atrium. After the formation of the secondary ostium, an additional secondary atrial septum (septum secundum) is formed, situated on the left side of the first septum.

The growing septum secundum becomes an ovally arching wall, closing the secondary ostium and forming its “roof” [6]. In the fetus, blood from the right atrium, where the pressure is higher, pushes the wall of the primary septum to the left, which ensures right-left flow at the level of the oval foramen. After birth, the pressure in the left atrium increases, leading to functional closure of the right-left flow. The condition for the proper development of the embryo and fetus is the maintenance of blood flow at the level of the atria, which is why in the prenatal period the defect in the atrial septum is not an anomaly.

Anatomical closure of the septum usually occurs a few days or weeks after birth. During the growth of the atria in the embryonic period, the pulmonary veins and the venous sinus are incorporated into their walls (of the left atrium and the right atrium, respectively).

During internal division at the level of the ventricle, a muscular projection grows upwards from the myocardium (a muscle fold located at the bottom of the primary common ventricle). Between the top of the muscular septum and the center of the primary heart tube, there remains for some time an opening connecting the formed right and left ventricles of the heart. At the next stage of development, this opening is covered by a membranous lamina, which eventually forms a membranous septum—the upper part of the interventricular septum. Around the 7th week of fetal life, the membranous septum joins the muscular septum, and the connection between the ventricles closes. By the 35th day after fertilization, the development of the embryo’s heart is complete [5].

The internal division of the heart tube at the level of the arterial trunk and the bulbus cordis separates the ascending aorta and the arterial trunk. In fetal life, both arteries remain connected by the ductus arteriosus (arising from the sixth left arch of the aorta). After the completion of the trunk divisions, the anteriorly located arterial trunk opens into the right ventricle, and the posteriorly located aorta ascends into the left ventricle.

The neural crest cells are involved in the formation of heart valves. From the thickening of the edges of the atrioventricular orifices, the tricuspid and mitral valves are formed. On the border of the arterial trunk and the bulbus cordis, transverse folds, the primordia of semilunar valves, are formed [47].

## 6. Development of the Cardiac Conduction System 

Specialized cells of the cardiac conduction system are formed from myocardial precursor cells. The cardiac conduction system develops from the SHF and from the neural crest cells. As the individual elements of the system are formed, they assume their correct position [5,23,36,37]. 

Along with the division and formation of the septa of the heart, rings of specialized conductive tissue are formed, which merge to form the atrioventricular node and branches of the conduction system as well as the Purkinje fibers. The onset of pacemaker development occurs on the 21st day after fertilization. The pacemaker (sinus node) is initially located in the caudal part of the left heart tube, from where it moves to the venous sinus [48].

Starting from the 5th week, i.e., from the moment the venous sinus is incorporated into the right atrium, the pacemaker is referred to as the sinoatrial node and is located in the right atrium, near the ostium of the superior vena cava. 

After a few days, an atrioventricular node is formed, located in the interventricular septum at the apex of the Koch triangle. The contractile stimuli are conducted from the sinoatrial node to the atrioventricular node through fibers of modified cardiomyocytes.

From the atrioventricular node, the atrioventricular bundle (bundle of His) is formed, whose left and right branches end in Purkinje fibers and conduct stimuli to the muscle of both ventricles [48]. 

The position and course of the conduction system in CHD are closely linked to the underlying congenital malformation. Although only subtle differences exist between the anatomy of the conduction axis for simple congenital heart lesions and normal anatomy, almost every CHD patient harbors some important anatomic variations [49]. 

## 7. Development of Large Blood Vessels

In addition to the heart arising from the heart tube, the arterial and venous systems play an important role in the development of the fetal circulatory system. The primary heart tube bifurcates into paired primary aortic arches, transforming into primary dorsal aortas, which then fuse together to form a single descending aorta. Parts of the dorsal aortas are connected to their abdominal segments by 6 paired aortic arches.

All aortic arches never occur together at the same time. The first pairs of the arches disappear before the last pair is formed. The first pair of aortic arches is the “mandibular” (1st) arch, which is involved in the formation of the maxillary artery and the internal carotid artery on each side. The second pair, “hyoid” (2nd) arch, participates in the formation of the small stapedial branch on each side. The third one is the “carotid” (3rd) arch that forms the common carotid arteries, the internal carotid artery (completed on each side with part of the dorsal aorta), and the external carotid artery. The fourth (4th) left “aortic” arch generates most of the aortic arch, completed by the left dorsal aorta as well as its continuation with the descending aorta, while the fourth right arterial arch transforms into the brachiocephalic trunk and the right subclavian artery (completed with part of the right dorsal aorta and the right seventh cervical intersegmental artery). The fifth (5th) aortic arch does not generate anything. The last (6th) aortic arch generates the bifurcation of the pulmonary trunk, the right pulmonary artery and the left pulmonary artery. The connection of the 6th left aortic arch with the left dorsal aorta remains, forming the ductus arteriosus or Botal duct. The fifth and sixth “pulmonary” arches (the most caudal ones) send their branches to the lungs [50,51]. Aberrant arch artery formation or remodeling leads to life-threatening congenital cardiovascular malformations, such as disruption of the aortic arch, cervical origin of arteries, and vascular rings. Severe defects associated with the morphogenesis of the pharyngeal arch arteries prevent adequate supply of oxygenated blood from the heart to the body [51].

The primary veins originate from the vascular plexus within the embryonic mesoderm. The venous vascular system undergoes transformations simultaneously with the development of arterial vessels. The embryonic venous system consists of paired veins, the so-called vitelline veins (carrying poorly oxygenated blood from the gallbladder), umbilical veins (carrying well-oxygenated blood from the chorion/placenta), and common cardinal veins (carrying poorly oxygenated blood from the fetus). The venous outlets reach the caudal branches of the heart tube. Some of them disappear, some of them transform, relatively often developing various deviations from the norm.

The development of the superior vena cava and the inferior vena cava is associated with the structures of the right horn of the venous sinus, and the left horn eventually transforms into the coronary sinus. The left vitelline vein regresses, and the right one gives rise to the inferior vena cava. The right umbilical vein disappears, and the left one produces the ductus venosus, which connects to the remaining disappearing section of the right umbilical vein, which is the primordium of the inferior vena cava. In the 8th week of the embryo’s life, the superior vena cava is also formed, its presence is preceded by the formation of the principal veins.

After the completion of internal divisions in the heart during the embryonic period, coronary circulation is formed. The coronary circulation develops from two primordia. The proximal primordium (pre-epicardial progenitor cells) is involved in the formation of vessels departing from the Valsalva sinuses (usually the aorta, rarely also the pulmonary trunk). The distal primordium is a forming network of vessels within the mesodermal angioblast of the developing myocardium. These primordia must fuse with each other. After the formation of the main coronary vessels from the angioblast within the atrioventricular sulci, they fuse with the projections of proximal primordia within the aortic sinuses by the invasion of endothelial cells into the aorta. Recent reports indicate that various sources of endothelial cells contribute to the mammalian embryonic coronary system. However, the specific fate and function of these different endothelial cell pools during coronary vascular morphogenesis is the subject of intense controversy [52].

Any irregularity in the position of the great vessels or the ventricles leads to defects in the fusion of angioblastic coronary vessels and projections departing from the Valsalva sinuses. Anomalies of the coronary arteries affect both their origin and distribution. Their identification is crucial in the preoperative assessment and surgical treatment of patients [53]. 

In parallel, although a little later in relation to the circulatory system, the lymphatic system develops. Three plexuses of lymphatic vessels are formed in the heart. Lymph from the subendocardial and myocardial plexus drains into the subepicardial plexus. Subsequently, larger lymphatic trunks are formed, running along the anterior and posterior interventricular sulcus [54]. Further development of the heart in utero after the completion of organogenesis consists mainly in myocyte growth. The cardiac development is completed between the 37th and 44th days of pregnancy.

## 8. New Heart Development Research Techniques

Despite the extensive information available on the different genetic, epigenetic, and molecular features of cardiogenesis, the etiology of some CHDs remains largely unknown. Recent research has revealed many aspects of cardiac morphogenesis that are important in the development of CHDs. This research is based on the new dynamic concept of heart development and the existence of two heart fields. 

A new vision for the analysis and diagnosis of congenital defects of the heart and great arteries, based on the development of in vitro models, has currently been designed to study embryonic development [55,56,57,58], and the design of ex vivo models [59,60] that mimic the embryonic microenvironment during heart development has contributed to a better understanding of important errors in cardiac morphogenesis, which may lead to several CHDs. The two main models are gastruloids and cardiac organoids. The first one is limited to investigation of cardiac development; the second one is becoming more and more helpful to simulate a functional beating heart. 

Three-dimensional (3D) gastruloids, aggregates of embryonic stem cells that recapitulate the key aspects of gastrula-stage embryos, have emerged as a powerful tool to study the early stages of mammalian post-implantation development in vitro. Owing to their tractable nature and the relative ease by which they can be generated in large numbers, 3D gastruloids provide an unparalleled opportunity to study normal and pathological embryogenesis from a bottom-up perspective and in a high throughput manner in humans [61]. Organoids, on the other hand, are miniature, 3D structures that originate from pluripotent stem cells and recapitulate cellular heterogeneity, structure, and behavior/function of cells of the original tissue, but under in vitro conditions. Therefore, they have the potential to model the functioning and physiology of human tissue more accurately than animal models or 2D models [62]. Organoids are extracted from pluripotent cells or tissues and are now becoming a versatile tool in the fields of cell biology, medicine, and pharmacy. The in vitro generation of cardiac organoids from induced stem cells (including human stem cells) has become an important tool in the study of cardiac tissue development. Unlike gastruloids, cardiomyocytes that differentiate into organoids show greater signs of maturation [56]. Drakhlis et al., described a method for recapitulating the first steps of human cardiogenesis in vitro. For this purpose, organoids referred to as heart-forming organoids were generated from human pluripotent cells. This methodology is a combination of the strategies of encapsulation with matrigel and modulation of the WNT pathway. It was found that organoids are composed of a myocardial layer covered with endocardial cells [58].

One of the main differences between gastruloids and cardiac organoids lies in the main signaling pathways that regulate their development. The regulation of signaling pathways and environmental stimuli can lead to the development of different cell lines within the organoid. The organoid and gastruloid models, along with tools for gene expression analysis, including single-cell RNA sequencing (scRNAseq), are an important advancement for understanding the processes that regulate the development of the heart at the cellular and molecular levels [3,59]. Moreover, the scRNAseq technology has confirmed the presence of different types of cardiogenic cells [59].

Lau and his colleagues generated an in vitro mouse embryo model with embryonic and extraembryonic lineages using exclusively embryonic stem cells and showed that the embryoids can undergo advanced development to late headfold stages. ScRNAseq demonstrates similarity between embryoids and natural mouse embryos [55].

Among the molecular mechanisms associated with CHDs that also include numerous transgenic lines should also be included. The findings of descriptive studies on human embryos and experimental studies on chicken, rat, and mouse embryos allow us to understand many of the mechanisms involved in correct heart formation and help us understand CHDs [55,63]. 

It has also been shown that in transgenic mice transgenic mice with cardiac-selective overexpression of NEXN developed an atrial septal defect (ASD) phenotype and that NEXN mutations in ASD patients inhibit GATA4 [64]. The genetically modified mouse is a good anatomical model for common cardiac malformations, which is a powerful experimental tool for understanding human CHD. Progress in the field of medicine is possible today on a large scale owing to the dynamic development of techniques that facilitate and expand the scope of experimentation and research. New frontier technologies should consider a new vision of cardiac development based on in vivo labeling and cell tracking for a better understanding of cardiac morphogenesis in health and disease conditions, which together provide a better understanding of the origins of CHDs.

Taking into account the rapid expansion of the gastruloid field, there is an increasing need for debates about the ethical aspects of human gastruloid models and for methods that improve the reproducibility of this emerging model system. The problem of scientific research on human embryos is at the heart of bioethical considerations and is also one of most difficult issues. The difficulty in indicating specific limitations in conducting experiments on human embryos results from the fact that the dispute on this topic is interdisciplinary in character, i.e., when analyzing the problem, the research results of the empirical sciences and humanities should be taken into account.

## 9. Impact of Genetic Factors on Heart Development 

Heart development is controlled by several overlapping morphogenetic systems that regulate intricate cardiac transcriptional networks [10,11]. 

The different structures of the heart come from different groups of cells and are controlled by different genes. The two major lineages that contribute to the developing heart are cardiomyocytes (CMs) and endocardial cells (ECCs) [10,65]. Cardiac development and formation is regulated by transcription factors (TFs) encoded by multiple genes. One of the genes responsible for the emergence of cardiac progenitor cell lines is the *Tinman* gene, first identified in the fruit fly (*Drosophila melanogaster*) [66,67,68,69]. In humans, there is a NKX2-5 gene homologous to the *Tinman* gene, and its abnormalities or absence lead to a defect in the structure of this organ. The expression of the NKX2-5 gene is limited exclusively to the cardiopoietic plaque and the surrounding endoderm, which is responsible for the formation of neuronal crests [69,70,71,72]. The transcription factors XIN, GATA, and MEF are also involved in this mechanism, and their activity is stimulated in particular by bone morphogenetic proteins (BMP-2, BMP-4, BMP-5, and BMP-7) produced by anterior endoderm cells [72,73,74,75,76]. The main stage in the development of the proper heart primordium is the expression of growth factor CR-1. This is because it determines the stimulation of the activity of the atrial natriuretic factor (ANF), MHC-2a and -2b genes, responsible for cell differentiation towards mesodermal cardiomyocytes [69,70,71,72,73,74,75,76,77]. 

The process of cardiac differentiation is controlled by fibroblast growth factors (FGFs), bone morphogenetic proteins (BMPs), Wnt proteins, and transcription factors TBX5, GATA4, and BAF60C [10,78,79,80,81]. The transcription factor GATA4 is essential for normal cardiac development [82,83]. In CMs, GATA4 regulates cardiac gene expression through collaborative interactions with other cardiac TFs, including NKX2-5, MEF2A, MEF2C, SRF, TBX5, and TEAD1 [84,85,86]. Less is known about the factors that cooperate with GATA4 to regulate gene expression in non-cardiomyocyte lineages such as endocardium cells. However, GATA4 has recently been reported to regulate endocardial development by interacting with the transcription factor ETS1 [83].

Cells of the primary and secondary cardiac fields (cardiogenic fields) are determined from the very beginning to produce right- and left-sided structures [5,22,87]. In the left-sided tube and its resulting structures, cells have a stable expression of the PITX-2C gene [88]. Within their walls, an internally located endocardial primordium and a superficial myocardial primordium are formed [89]. They are composed of endocardial endothelial precursor cells and myocardial precursor cells, respectively, which, unlike the previous cells, demonstrate stable expression of the MESP-1 gene and transient of the PCMF-1 gene [69]. The precursor endocardial endothelial cells are characterized by constant gene expression for multiple transcription and growth factors (i.e., VEGF-R2, TIE-2, PECAM) and proteins (i.e., thrombomodulin, actin, fibrillin 2) [77,90,91]. 

At the same time, as a result of bending the embryo’s body walls and closing the foregut tube, both heart tubes come closer to each other and then fuse, which results in the formation of the so-called single tube heart [92]. The fusion of the heart tubes is accompanied by strong expression of the VMHC-1 gene of cardiac myosin heavy chains [93]. At a later time, there is also expression of the MLC-2V, MLC-2A, and A-MHC genes, encoding light and heavy myosin chains in ventricular and atrial cardiomyocytes. Activation of the first of these genes is dependent on high expression of NKX2-5 and on the Carp factor, and the GATA protein associated with it [94,95,96]. A constant expression of the ANF gene also appears, especially in atrial cardiomyocytes [93]. When the process is completed, the expression of the NKX2-5 gene in cardiomyocytes ceases and persists only in the endocardial cells of the right ventricle and the right atrium [74,76,97]. 

The process of bending and twisting individual parts of the heart tube is regulated genetically, most likely by the uneven distribution of cells with high expression of the XIN gene [73,98]. Such cardiomyocytes predominate on the right side of the organ, and the transcriptional activity of the gene is mainly stimulated by BMP-2. Myocyte enhancer factor 2 (MEF-2) also plays an important role in this process [77]. The expression of the gene encoding it is typical during the rotation period and persists until the final formation of the four-chamber structure [99]. This is most likely due to the binding of the encoded protein to desmin precursors and atrial- or ventricular-specific myosin light chain-2 (MLC-2) promoters [100]. Tessadori et al. conclude that cardiac looping involves twisting of the chambers around the atrioventricular canal, which requires correct tissue patterning by TBX5A [101]. 

At the further stage of differentiation of the chambers and walls of the heart (cardiac structures) during the development of atrial formation on the border of the primary atrium and the primary ventricle—within the so-called atrioventricular canal—a gelatinous substance accumulates, forming elongated endocardial pads demonstrating high expression of PITX-2C [102,103]. Within the endocardial pads, there is also a high expression of neuregulin and its receptor (ErbB-3) genes, which results in stimulation of the local development of mesenchymal cells [104]. This process depends mainly on the BMP-5 protein synthesized by cardiomyocytes [87]. The uneven development of the atrial endocardium is most probably due to the early, albeit phasic, inhibition of MLC-2v expression while maintaining constant expression of MLC-2a, a-MHC, and ANF [10,90]. The atrial myocardium is also characterized by the presence of fibroblastic growth factor receptors (FGFR-2) [105].

In ventricular development, as a result of rotation of the primary ventricle, the muscular part of the interventricular septum is formed [5]. The septum-determined cells highly express MSX-2 compared to ventricular wall cardiomyocytes [72]. The anteriorly located right ventricle becomes connected to the atrium by further metamorphoses and displacement of endocardial pads, and its cardiomyocytes demonstrate high expression of hHAND and MEF-2. In the left ventricular endocardium, eHAND expression predominates [70,106]. Multiple transcription factors have been shown to regulate cardiac chamber morphogenesis, including the T-box transcription factors TBX2, TBX3, TBX5, and TBX20, as well as NKX2-5, GATA4, CITED1, and IRX4, IRX1/IRX3/IRX5. TBX5 and TBX20 act in combination with NKX2-5 and GATA4 to promote specification of chamber myocardium [107]. These processes lead to the final formation of both atrioventricular orifices and the mitral and tricuspid valves that close them, respectively, on the left and right sides [10,108]. 

Along with the formation of the endocardium, its internal differentiation occurs. Neuralgine secreted by endocardial endothelial cells and stimulated by HGF-2, BMP-2, and -4 is responsible for the formation of myocardial trabeculae [77,89,109]. Retinoic acid also influences this, which stimulates cell division of the deep endocardium while inhibiting their differentiation process via RXRα and RXRβ receptors [90,109,110]. Convergent activity is also demonstrated by c-Myc [109,111], serum response factor (SRF) [66], VCAM-1, and N- and T-cadherin [112]. 

The signal for the development of vascular processes comes from the epicardium and is transmitted by growth factors vascular endothelial growth factor (VEGF), fibroblast growth factor (FGF), and platelet-derived growth factor (PDGF) [113] and by the complex signaling pathway network consisting of TGF-β/Smad, BMP, Wnt/β-catenin, Notch, and Smad-independent TGF-β signaling pathways, which together induce the expression of transcription factors such as Snail, Slug, and Twist and promote or inhibit EMT [45,114,115,116,117]. The factors that control the formation of the coronary arteries are VEGF, PDGF, and substances such as erythropoietin and retinoic acid [113]. 

The proper formation of the atrioventricular node depends on transcription factors, which play many roles during the development of the heart. This group includes NKX2-5, TBX5, and GATA4 factors [83,118]. In the cells that give rise to the sinus node, the fetal TBX18 gene is expressed, while in the cells that give rise to the atrioventricular node and the Purkinje fiber system, the NKX2-5 transcription factor expression has been found [118].

## 10. Etiology of Congenital Heart Defects (CHDs)

**Figure 5:**
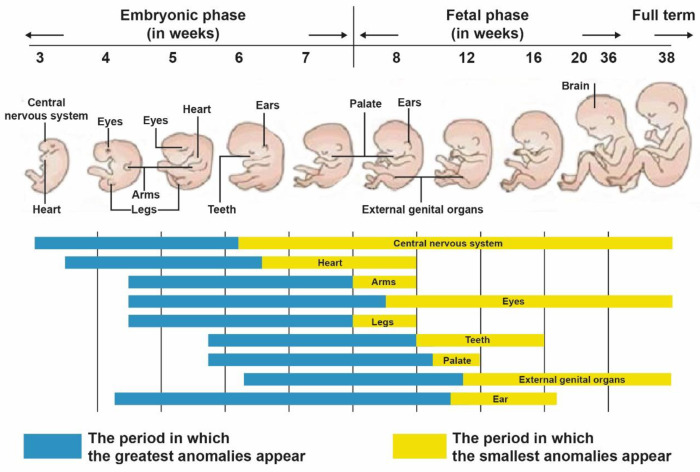
The diagram shows the stages of development in which the greatest and smallest anomalies in selected structures in humans occur in the embryonic and fetal phases. This figure was modified and adapted from Larsen [119].

The development of most hearts takes place not in neonates but in the early prenatal period. It should be noted that the risk of CHDs is greater when the future mother is unaware of the fact that she is pregnant. As quoted above, the formation of the first embryonic structures in heart development occurs already in the first three weeks after fertilization (Figure 4 and Figure 5). At this stage of embryonic development, not every woman realizes that she is pregnant. Therefore, the future mothers are likely to engage in risky behavior relatively often, and that is the reason why it is often difficult to show what the relationship is and what may be the direct cause of the defect. There is scientific evidence confirming the correlations between CHDs and cardiovascular diseases, other birth defects, genetic links, neurological disorders, maternal age, and others listed later in the manuscript. During the past decades, a consensus has emerged that both genetic and environmental factors are related to the occurrence of CHDs [13,14,15,16]. Table 1 presents some non-genetic factors contributing to the induction of CHDs.

### 10.1. Prevalence and Regional Variation of CHDs

CHDs are the most frequent of the major congenital anomalies, representing a major global health problem, and are responsible for one of the highest mortalities in newborns after birth, especially if delivered preterm and twin pregnancies [120,121,122]. Occurs in approximately 1% of all liveborn infants [120]. CHD birth prevalence worldwide and over time is suggested to vary [120,123,124]. Indeed, 28% of all major congenital defects consist of heart defects [121,125]. Reported total CHD birth prevalence increased substantially over time, from 0.6 per 1000 live births in 1930 to 1934 to 9.1 per 1000 live births after 1995. Over the last 15 years, stabilization occurred, corresponding to 1.35 million newborns with CHD every year [120]. The estimate of 8 per 1000 live births is generally accepted as the best approximation. However, this estimate is perhaps inaccurate and does not take into consideration regional differences [121,126,127]. 

Regional variation exists in the prevalence of CHDs [120,123,128]. Asia has reported the highest CHD birth prevalence, with 9.3 per 1000 live births [120]. The reported total CHD birth prevalence in Europe was significantly higher than in North America—8.2 per 1000 live births vs. 6.9 per 1000 live births [120]. The burden of CHD in India, Asia, and Africa is likely to be the largest among all nations in the world simply because of the fact that there are more children born in these countries than anywhere else [128,129]. The observed differences may also be of genetic, environmental, socioeconomical, or ethnic origin. 

### 10.2. Maternal Factors

Reaching reproductive age in CHD patients revealed the possibility of transmitting cardiac anatomical anomalies to their offspring [130,131]. Based on data for cases of CHD from 24 European population-based registries, evidence of a positive association between maternal age and the total prevalence of CHD for younger (≤24 year-old) and older (35–44 year-old) mothers was observed. The research results suggest that young maternal age (≤24 years) is a factor associated with severe CHD phenotypes, while a positive association between advanced maternal age (35–44 years) and mild CHD phenotypes was observed [131]. 

The increase in the risk of CHDs with maternal age is associated with biological processes such as aging of egg cells, decreased sperm quality in men, changes in gene quality, and reduced cell repair capacity. Additionally, older women are more likely to have comorbidities that can affect the health of their offspring.

The severity of CHDs is determined by the stage of gestation during which development is disturbed, the extent of impairment, and the embryo’s ability to compensate for the defect [132]. CHDs can be caused by defects occurring during embryogenesis between the 3rd and 8th weeks of gestation when the main structures of the cardiovascular system develop. However, the origin of CHDs is not fully understood.

There is ample evidence of the influence of genetic, epigenetic, and environmental factors associated with the development of CHDs.

### 10.3. Genetic Factors

Dr. John Maurice Hardman Campbell published the first paper on CHD genetics in 1949, and since then variants in hundreds of genes have been identified that may cause or contribute to CHDs [133,134,135,136,137]. Genetic factors are observed in approximately 10–15% of cases, including the specific loci involved in familial cases of CHD [3,11,19,138]. 

It has been revealed that the prevalence of CHD in first-degree relatives (22.0%) was significantly higher than that in second-degree relatives (3.4%). Additionally, the recurrence rates of CHD in families in which the patient’s mother or sister had CHD were significantly higher than in cases with the father or brother having CHD. In the sets of familial pedigrees analyzed, the concordance of affected by CHD in identical and dizygotic twins affected was 94.4% and 33.3%, respectively. These authors also showed that threatened abortion in early pregnancy was associated with familial CHD [139].

Currently, the most widely known genetic defects are autosomal single- and multi-gene mutations that cause the loss or gain of function of a particular protein, predominantly transcription factors, and certain chromosomal abnormalities such as trisomies 13, 15, 18, 21, and 22q11.2 deletion [140,141]. With recent advancement in next-generation sequencing technologies, a genetic abnormality—defined as chromosomal aneuploidy, a pathogenic chromosomal copy number, or a single gene variant—is possible to be detected in approximately 1/3 of CHD cases [142].

### 10.4. The Influence of Environmental Factors

In 85–90% of CHD cases, environmental exposure is the most widely accepted etiology [3,11,19]. The most serious congenital heart defects in humans occur in the offspring of mothers with abnormal folic acid supply and metabolism [143,144]. They often coexist with neural tube abnormalities [145]. As a result of folic acid deficiency during pregnancy, the migration of neural tube cells to the heart primordium is disturbed, which contributes to the development of defects of the conus and the neural tube. The most common defects of the conus and the arterial trunk include transposition of the great arteries (TGA), tetralogy of Fallot, and common arterial trunk [13,146,147]. The periconceptional use of folic acid reduces the risk of CHDs, including TGA, and its intake is recommended as a protective factor against a large spectrum of congenital malformations [148]. 

Important risk factors include congenital rubella syndrome, diabetes during pregnancy [149,150], and other maternal factors such as obesity and lifestyle [151], bronchial asthma [152], as well as maternal nicotinism and alcoholism and the use of designer drugs [153,154]. Most epidemiological studies also mention exposure to infectious factors, especially bacterial [108] and viral in the first trimester of pregnancy [155]. 

Among the chemical agents, the classic teratogenic teratogens are distinguished. One of them is retinoic acid, which models the individual stages of organ development. The cells of the secondary heart field are particularly sensitive to fluctuations in acid concentration. As a result of its absence, they do not migrate to the proper outflow pathways but move to the right, causing the formation of a heart defect with a double-outlet right ventricle [156,157]. There are also disturbances in the formation of the lesser curvature of the heart, and therefore the aorta and the pulmonary trunk arising from this part of the organ cannot rotate properly, which leads to an abnormal, right-sided position of the aorta, referred to as dextroposition [146]. 

An excessive amount of acid also contributes to impaired morphogenesis and increases the risk of, i.e., double right ventricular outflow, transposition of the great arteries, and pathological ventricular septal defect [158]. Also, exposure to vitamin A retinol has a teratogenic effect on the heart [159]. Other environmental factors teratogenic to the outflow pathway area include nitrophene and atmospheric pollution by chlorinated hydrocarbons and pesticides [160], as well as exposure to dietary arsenic during the year before pregnancy [161]. 

The most consistent method to reproduce TGA in animal models is treating pregnant mice with retinoic acid, which is the active metabolite of vitamin A, or with retinoic acid inhibitors [162,163]. As the developing cardiovascular system is particularly sensitive to different levels of retinoic acid, it appears that its administration at different time points of pregnancy produces different cardiac phenotypes. In fact, mouse experiments produced not only cases of TGA with ventricular D-loop, but also some cases of TGA with ventricular L-loop (ccTGA) [164]. 

**Table 1:** Exposures associated with definite or possible risk of offspring with CHD. Specific CHD are listed for exposures shown to produce a known defect. The table was modified based on the work of Jenkins et al. [13] and Mahler et al. [165].

	Known Defect(s)
**Maternal Illness**	
Phenylketonuria	
Pregestational diabetes	Conotruncal defects
	Laterality and looping
	Dextro-looped transposition of the great arteries
	Atrioventricular septal defect
	Septal defects
	Hypoplastic left heart syndrome
	Outflow tract defects
	Patent ductus arteriosus
Febrile illness	Conotruncal defects
	Right-sided obstructive defects
	Tricuspid atresia
	Left-sided obstructive defects
	Aortic coarctation
	Ventricular septal defects
Influenza	Conotruncal defects
	Dextro-looped transposition of the great arteries
	Right-sided obstructive defects
	Left-sided obstructive defects
	Aortic coarctation
	Ventricular septal defects
	Dextro-looped transposition of the great arteries with intact ventricular septum
	Tricuspid atresia
Maternal rubella	Ventricular septal defects
	Patent ductus arteriosus
	Pulmonary valve abnormalities
	Peripheral pulmonic stenosis
Epilepsy	
**Maternal therapeutic drug exposure**	
Anticonvulsants	
Indomethacin tocolysis	Patent ductus arteriosus
Ibuprofen	Dextro-looped transposition of the great arteries
	Ventricular septal defects
	Bicuspid aortic valve
Sulfasalazine	
Thalidomide	
Trimethoprim-sulfonamide	
**Maternal nontherapeutic drug exposure**	
Maternal vitamin A	Outflow tract defects
	Cranial neural crest defects (cardiac and noncardiac)
	Pulmonic stenosis
Marijuana	Ventricular septal defects
	Ebstein’s anomaly
**Maternal environmental exposure**	
Organic solvents	Conotruncal defects
	Hypoplastic left heart syndrome
	Aortic coarctation
	Pulmonic stenosis
	Dextro-looped transposition of the great arteries with intact ventricular septum
	Tetralogy of Fallot
	Total anomalous pulmonary venous return
	Atrioventricular septal defect
	Ebstein’s anomaly
	Ventricular septal defects

Anomalies of cardiac morphology can also be a consequence of medications used [165], such as xenobiotics, which include, i.a., cyclooxygenase inhibitors, taken in infections to lower body temperature [13,166], antiepileptic [167], and hormonal drugs [168]. Among the physical factors, exposure to ionizing radiation has an impact [169]. Table 1 lists some known cardiac teratogens and the specific CHD they cause. 

Among other risk factors for CHD, in vitro fertilization (IVF) should also be mentioned [170]. It has been proven that the intracytoplasmic sperm injection (ICSI) fertilization technique interferes with the process of genetic stigmatization. CHD was demonstrated to occur in 1.3% of children conceived by IVF/ICSI vs. 0.68% of children from natural conception [171]. Another study assessed the prevalence of CHD at 7.1% in children conceived in vitro, 9.9% in children conceived by ICSI, and 5.7% in natural pregnancies [172]. 

### 10.5. Epigenetic Processes

Epigenetic changes triggered by adverse environmental factors have been reported to directly influence the phenotype and translate into pathological conditions. Thus, epigenetic processes, such as DNA methylation, histone modifications, and non-coding RNA activity, are involved in the development of the heart [11,18,173]. They modulate chromatin accessibility and allow the transition from a transcriptionally active chromatin state (euchromatin) to an inactive chromatin state [174,175]. Furthermore, the ability of single epigenetic modifications to control the expression of several genes has implicated their dysregulation in many polygenic diseases, including CHDs. It has been demonstrated that many of the histone-modifying enzymes that regulate remodeling in disease processes also play a role in normal heart development [176]. Epigenetic regulators such as p300, SMYD1, EZH2, and BRG1, as well as some transcription factors, GATA 4, NKX2-5, MEF2, and HAND2, play an important role both in the health processes and in the development of CHDs [11,177,178]. 

Non-coding RNAs involved in the control of gene expression primarily include long non-coding RNA (lncRNA) and microRNA (miRNA or miR). miRNAs are often tissue-specific and tend to regulate gene expression by modulating feedback loops. It has been shown that miR-1-1 and miR-1-2 are expressed specifically in cardiac progenitor cells and that Mef2 upregulates miR-1-1 and miR-1-2 expression, which suppress Hand2 and class II HDAC translation [179,180]. Downregulated HDAC4 upregulates Mef2 in a positive feedback loop, and the control of Hand2 maintains a careful balance between cardiac cell differentiation and expansion [181]. Furthermore, miRNAs have been shown to be essential for cardiomyocyte proliferation, maturation, and pathogenic remodeling, making them promising candidates for the preservation and correction of heart function in patients with structural heart defects. miRNAs and DNA methylation are also promising biomarkers for the prenatal and postnatal diagnosis and stratification of patients with CHDs. miRNA therapies for CHDs are particularly attractive compared to other epigenetic pathways because of the vast arsenal of genes that they regulate, as well as their gene and tissue specificity [11].

The etiology of CHD is becoming increasingly defined based on prior epidemiologic studies that supported the importance of genetic contributors and technological advances in human genome analysis. Overall, there are still many unknowns surrounding the genetic and environmental contributions to CHD, and this area should be the focus of major research efforts in the future.

## 11. Sequential Segmental Analysis of the Heart

The most popular diagnostic paradigm and method used in the world for description of the congenitally malformed heart is sequential segmental analysis of the heart. This method was established by Prof. Richard van Praagh and modified by Prof. Robert Anderson and Prof. Anton Becker [182]. 

They have described a nomenclature for congenital heart disease employing sequential chamber localization. It links together the atrial, ventricular, and arterial segments of the heart and then enables tabulation of associated anomalies. The assessment of atrial morphology is postulated as the first diagnostic step.

The atrial segment of the heart can exist as situs solitus (S), situs inversus (I), or situs ambiguus (A). In a normal heart, where the morphologically right atrium is located correctly on the right side and the morphologically left atrium is located on the left side, this situation is referred to as atrial situs solitus. The reverse position of the atria, when the morphologically right atrium is located on the left side, and the morphologically left one on the right, is called situs inversus of the atria. Situs inversus is the mirror image of situs solitus. In heterotaxy syndromes, both atria have a similar, almost identical structure, i.e., two morphologically right atria or both morphologically left atria, which we define as situs ambiguus of the atria, or right or left isomerism [183]. 

The next step is to assess the atrioventricular connections to determine which ventricle each atrium communicates with, as well as the morphology of the atrioventricular valves. In a normal heart, this connection is called a concordant biventricular atrioventricular connection. A discordant connection is understood as a situation when the morphologically right atrium connects to the morphologically left ventricle and the morphologically left atrium connects to the morphologically right ventricle [182].

**Figure 6:**
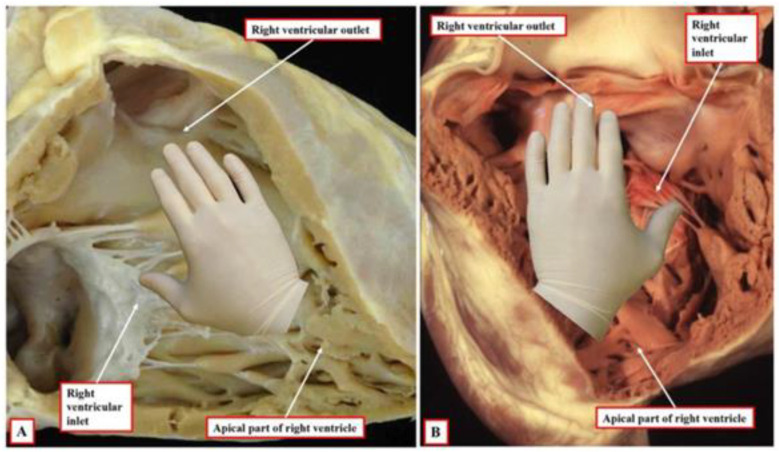
The images show the essence of the patterns of ventricular topology as seen in postnatal hearts. Panel (**A**) shows a normal right ventricle, which accepts on its septal surface only the palmar surface of the right hand when the thumb is placed in the inlet, with the fingers extending into the outlet. Panel (**B**) shows the left-sided morphologically right ventricle from a heart with congenitally corrected transposition. This right ventricle accepts only the palmar surface of the left hand. The figure was taken from Crucean et al. [184] and adapted under the terms of the Creative Commons Attribution License (CC BY).

The position of the ventricles is described in a similar way to that of the atria, but we do not use the term situs here. There are two basic types of ventricular relations (situs): 1. solitus (D-loop), 2. inversus (L-loop). The criterion proposed by Prof. Anderson is defining the topology of the ventricles as either the right- (correct) or left-hand type (Figure 6). In the D-loop, the right ventricle corresponds to the right hand (it is “right-handed”). It consists in the fact that after placing the right hand on the septal wall of the morphologically right ventricle, the thumb should indicate the inflow route, and the other fingers should indicate the outflow route from the right ventricle. In a normal heart, this configuration corresponds to the right hand. The left ventricle can be identified using a similar position of the left hand (“left-handed”). The topology of left-hand type (L-loop) ventricles is referred to as ventricular inversion [182,184]. 

The assessment of the cardiac septum is based on the analysis of the possible defects in its individual parts together with the determination of the defect diameter.

The assessment of the atrioventricular valves involves examining not only the number of leaflets, but also the number of rings and their relation to the septum and valve apparatus.

The configuration of each heart can be represented by a cardiotype, i.e., a combination of three symbols corresponding to the main segments of the heart—atria, ventricles, and large vessels—usually enclosed in curly brackets. The symbols denote the situs (alignment) and chiral characteristics of the individual segments, and their order is consistent with the direction of blood flow. The anatomy of the connecting segments (the atrioventricular canal, the cone) and the type of connection between the segments, as well as the concomitant defects are marked outside the bracket. The relationships between the segments of the heart denote their position relative to each other in space, but they do not denote the way they are connected to each other.

Van Praagh introduced a method of abbreviated recording of the segmental morphology of the heart, which is still used in many centers around the world [185,186]. 

Normal heart anatomy is described as the {S, D, S} cardiotype, which denotes:

1. {S, -, -}—situs solitus—Normal visceroatrial relationship, i.e., the presence of both venae cavae and the right atrium on the right side, whereas the left atrium is on the left side. The right atrium is “right-handed”, and the left one is “left-handed”.
2. {-, D, -}—D-loop, i.e., right-handed cardiac loop, the right ventricle is “right-handed” and located on the right and anteriorly in relation to the left ventricle. The left ventricle is “left-handed” and situated to the left and inferiorly to the right ventricle due to the right-sided looping of the embryonal heart tube.
3. {-, -, S}—normal position of the large blood vessels, i.e., the aorta located posteriorly and to the right of the pulmonary artery trunk. The ventriculovascular junction is concordant, and the cone is located under the pulmonary artery.
4. The {l, L, l} cardiotype denotes also a normal heart, but in situs inversus (mirror image).

{S, L, L} TGA is a physiologically corrected transposition with a normal visceral-atrial relationship, an L-type ventricular loop, and the position of the aorta on the left side of the pulmonary trunk. In this case, there is atrioventricular and ventriculovascular discordance.

The correct atrioventricular alignment is presented in the case of the following cardiotypes: {S, D, -} and {l, L, -}. Incorrect atrioventricular alignment is marked with the symbols: {S, L, -} and {l, D, -}. According to Richard von Praagh, the three main cardiac segments can be positioned differently and fused together by two additional connecting segments, i.e., the atrioventricular canal and the cone (infundibulum). The atria are not connected with the ventricles to form a morphological whole (with the exception of the bundle of His), since they are separated by fibrous elements of the atrioventricular canal that isolate these chambers from each other electrically. Similarly, the ventricles do not connect directly to the large vessels, but through a connecting segment—a cone that can be a fully developed muscular structure, or a fibrous remnant [186,187]. 

## 12. Ventriculoarterial Relationship 

The next stage of the sequential segmental analysis is to determine the site of departure of the great vessels of the heart base, but also their location in relation to each other as well as the vessels themselves in terms of their course, abnormal diameter, interruption of continuity, etc. Ventriculoarterial connection variants can be (a) normal; (b) transposition; (c) double outlet ventricle; or (d) single arterial trunk. The associated anomalies are categorized in terms of venous return, atria, atrioventricular junction, ventricles, and great arteries [188].

The ventriculovascular relationship may be normal (situs solitus) {S, D, S} or normal fully inverted (situs inversus) {l, L, l} (the left ventricle connects to the aorta and the right ventricle to the pulmonary trunk). D-transpositon, D-TGA {S, D, D} or L-transposition, L-TGA {S, L, L} large vessels means the junction of the left ventricle with the trunk of the pulmonary artery, and the right ventricle with the aorta. Double-outlet right ventricle (DORV) may occur in different configurations, e.g., DORV {S, D, D}, DORV {S, L, L}, or DORV {S, D, L}.

{S, D, D} TGA means the transposition of the great vessels with the correct visceroatrial relationship (S-solitus), the D type ventricular loop (D-loop), and the position of the aorta in relation to the pulmonary trunk in position D (the aorta is located on the right side in relation to the pulmonary trunk). The symbol outside the brackets (TGA) describes the type of ventriculovascular junction and denotes the transposition of the great trunks (ventriculoarterial discordance), i.e., the junction of the pulmonary trunk with the left ventricle and the aorta with the right ventricle. 

Each segmental description must be supplemented by an analysis of accompanying defects. Only a complete description allows a complete and comprehensible analysis of the anatomy of a heart with a congenital defect.

## 13. Conclusions

The development of the cardiovascular system is determined by many factors, both intrinsic and extrinsic. The development of the heart involves complex biochemical signals, interactions, and specification of myocardial progenitor cells and heart tube looping. Cell populations originating from outside the primary heart field and the linear heart tube of the embryo contribute to the development of the mature heart and modulate cardiac morphogenesis. These cell populations include neural crest cells, cells derived from the second heart field, and epicardial cells.

Disturbances of any stage of organogenesis may lead to various congenital heart diseases and defects that could be initiated by various genetic, epigenetic, or environmental factors. The transition from the fetal to neonatal circulation is considered to be a period of intricate anatomical and physiological changes in the cardiovascular system. New techniques for studying cardiac development that allow for the identification of abnormalities in early cardiogenesis and the cardiovascular system physiology in the period of transition from fetal to neonatal life are essential for the immediate and long-term cardiovascular health risk of neonates. 

The implications of the transformation of a fetal heart into a mature heart remain the areas of research that are likely to improve our understanding of congenital heart defects in the future. The research on the regulation of gene expression programs will lead to the proposal of new therapeutic targets for their treatment. In the coming years, further advances in knowledge about the development of the heart are likely to have an even greater impact on the classification and treatment of congenital heart defects.

## Data Availability

Data sharing is not applicable to this article.

## Abbreviations

ANF	atrial natriuretic factor
AV	atrioventricular
BMPs	bone morphogenetic proteins
cc-TGA	congenitally corrected transposition of the great arteries
CHD	congenital heart defect
CM	cardiomyocytes
CNCCs	neural crest cells
DNA	deoxyribonucleic acid
D-TGA	transposition of the great arteries
DORV	double outlet right ventricle
ECCs	endocardial cells
EMT	epithelial-mesenchymal transformation
FGF	fibroblast growth factor
FHF	first heart field
LV	left ventricle
miRNA	microRNA
PDGF	platelet derived growth factor
RA	right atrium
RNA	ribonucleic acid
RV	right ventricle
SHF	second heart field
SA	situs ambiguus
SI	situs inversus
SS	situs solitus
TF	transcription factor
TGA	transposition of the great arteries
TGF-β	transforming growth factor beta
VA	ventriculoarterial
VEGF	vascular endothelial growth factor
